# Mini marvels: superhero engagement across early childhood

**DOI:** 10.3389/fpsyg.2025.1537115

**Published:** 2025-05-30

**Authors:** Sarah M. Coyne, Sarah Ashby, Rachel J. Munk, Hailey G. Holmgren, Jane Shawcroft, Rebecca Densley, Tanya Austin, Kennedy Banks, Megan Van Alfen

**Affiliations:** ^1^School of Family Life, Brigham Young University, Provo, UT, United States; ^2^Department of Human Ecology, University of Alberta, Edmonton, AB, Canada; ^3^Department of Communications, University of California, Davis, Davis, CA, United States; ^4^Trinity University, San Antonio, TX, United States

**Keywords:** superhero, media, aggression, children, defending, prosocial behavior

## Abstract

**Introduction:**

Superhero engagement is common in early childhood, particularly among boys, and tends to be related to negative outcomes, such as aggression. However, most research lumps all types of superhero engagement together and is conducted over a relatively short space of time. The current study is a five-year longitudinal study of four different types of superhero engagement (identification, toys, dress up, and media).

**Method:**

Participants included 430 children (*M* age = 29.17 months at Wave 1) and their primary caregivers who were asked questions about superhero engagement and social behavior once a year for 5 years.

**Results:**

Overall, superhero engagement was relatively high in early childhood and showed distinct trajectories depending on the type of engagement, with the most rapid growth occurring in superhero media. Boys and those who viewed high levels of television were more likely to have higher levels of superhero engagement. Additionally, early high identification with superheroes and playing with superhero toys and moderate but increasing superhero media predicted higher levels of aggression and aggressive defending over time. Dressing up as superheroes was related to very few outcomes during early childhood.

**Discussion:**

Overall, this study has implications for parents, educators, and creators of superhero media and merchandise.

## Mini marvels: superhero engagement across early childhood

From a young age, many children are inundated with superhero culture—via screen media, books, toys and action figures, and dress-up costumes (O'Connel, 2019). These fictional characters share common characteristics including a pendant for justice, fantastical abilities (e.g., strength, flight, or mind-reading), and a tendency to conquer all in the face of adversity. Superheroes often exhibit teamwork, positive relationships with others, and helping those in need —attributes that could make them positive role models for children. However, many superheroes also exhibit aggressive or violent behavior, typically to protect the innocent or uphold moral values of justice, truth, fairness, freedom, or social order (Bauer et al., 2017). Some scholars have questioned whether these depictions of violence are harmful for children, outweighing the positive attributes modeled by superheroes (e.g., Morgan and Schwebel, 2024). Indeed, research suggests that exposure to superhero media and superhero identification relate to higher levels of aggression (Coyne et al., 2014) and risk-taking behaviors in children (Morgan and Schwebel, 2024).

Although most superhero violence is depicted as benevolent, it is unclear whether children can discern this distinction or apply it to their own social interactions. For example, a recent longitudinal study found that superhero engagement was linked with higher physical and relational aggression, but not with defending or prosocial behaviors (Coyne et al., 2017). This underscores the need to further explore associations between superhero engagement and aggressive defending behaviors. Further, no studies to our knowledge have examined how engagement with superheroes develops across the course of early childhood and how this might be linked to relational processes. Thus, the aim of the current study is to examine the differential growth of superhero engagement (identification, playing with toys, dressing up, and viewing superhero media) across a 5-year period (ages 2.5 to 6.5 years old) and how these are related to aggression, prosocial behavior, and defending behavior.

### Superhero engagement

*Superhero engagement* refers to the frequency and salience of children's engagement with superhero characters or superhero-related content (Coyne et al., 2014). In this paper we address four components of child superhero engagement: (1) identifying with superheroes (i.e., seeing them as a role model, mimicking their behavior or characteristics), (2) playing with superhero-themed toys, (3) viewing superhero-related media content (i.e., movies, TV shows), and (4) dressing up as a superhero for pretend play. According to social learning theory (Bandura, 1969) and sociocultural theory (Vygotsky, 1978), engaging with superheroes may influence children's behaviors, including aggression, defending behavior, and prosocial behavior. For example, identifying with superheroes might shape children's normative beliefs and expectations about behavior. Playing with superhero toys or dressing up allows children to act out these behaviors, which may, in turn, affect their actions in other settings. Additionally, superhero media offers strong role models that children may imitate in their interactions with others. These behaviors are often explored as part of the larger construct of superhero engagement (e.g., Coyne et al., 2014), but are unique behaviors (e.g., Bauer et al., 2017). For example, some children may consume superhero media and develop a strong identification with the characters without engaging in related play, such as using toys or dressing up. Conversely, other children may possess superhero-themed toys but show little engagement with superhero narratives through other forms of interaction. Thus, it is possible that certain types of superhero engagement emerge and grow distinctly from other types and may differentially predict developmental outcomes. We review each type of superhero engagement in turn.

#### Superhero identification

Children's identification with others has long been regarded as central to their development and socialization as it allows them to see the world from another's point of view, resulting in expanded skills and perspectives (for a theoretical review see Cohen, 2001). Bandura (1969, p. 249) social learning theory suggests that children are more likely to mimic the behaviors of models whom they admire and identify with, and media scholars extend this to identification with media characters wherein audience members experience, “feelings of affinity, friendship, similarity, and liking of media characters.” Early media studies (e.g., Huesmann et al., 1984; Maccoby and Wilson, 1957) point to identification with media characters as a central mechanism for learning from media and subsequent imitation of characters' behaviors.

More recently, scholars examining the influence of superhero media posit that superheroes are salient models who often have desirable and romanticized characteristics including physical attractiveness and superhuman powers (Harriger et al., 2022; Morgan and Schwebel, 2024). Thus, one way that children can engage with superheroes is to identify with them—in other words, they like and/or admire a hero and engage in vicarious experience (e.g., perspective taking) through that character. The process of superhero identification likely evolves over time, with children internalizing superhero values and behaviors differently across childhood (Anderson et al., 2010; Coyne et al., 2017; Fraser et al., 2012). We expect that superhero identification will intensify as children mature, aligning with their developmental understanding of and desire for strength and heroism.

#### Superhero toys

The types of toys children engage with often reflect their interests and developmental stage (Ramdaeni et al., 2020). Superhero-themed toys, weapons, and items from superhero shows are commonly used in sociodramatic play, which can involve forms of aggression, weapon play, and defending behaviors (Coyne et al., 2014). However, as children mature, their preferences tend to shift with a greater focus on age-appropriate activities or a transition toward technology-based entertainment rather than handheld toys (Wang et al., 2022). Consequently, we anticipate that the frequency superhero toy play will decline as children grow older, as a reflection of their changing developmental needs and interests.

#### Dressing up

Sociodramatic play, including imaginative play and role-playing scenarios (Dinham and Chalk, 2018) is a central aspect of early childhood development. Wearing costumes is common in childhood, with costumes becoming more gendered over time (Dinella, 2017). Many young children engage in sociodramatic play by dressing up as their preferred superheroes, either during playtime or as part of their everyday outfits (Coyne et al., 2014). Sociodramatic play allows children to explore various roles and identities, and to perhaps mirror activities and behaviors of peers, parents, siblings, or media characters (e.g., superheroes). Vygotsky's (1978) sociocultural theory helps to explain what children may gain from superhero play, by suggesting that children may model positive helping and aggressive defending behaviors seen in superhero media, as well as absorb the moral lessons within superhero media (e.g., the importance of helping others, using violence to help others). However, as children grow older and their developmental needs and cognitive abilities change, we predict that the frequency of dressing up as superheroes will decrease as children age.

#### Superhero media

From an early age, children are exposed to movies and TV shows, many of which prominently feature superheroes. Superhero media engagement begins with children's television shows including *Spidey and His Amazing Friends, PJ Masks, and The Powerpuff Girls*, among many others, and extends into teenage and adult-focused media including the expansive Marvel Universe and the DC franchise. Superhero media is one of the few media genres to span such a wide age range, with stories and characters that evolve across platforms and age groups (Martin, 2023). For example, *Spidey and His Amazing Friends* are targeted toward preschool age children, while *Spiderman: Across the Spiderverse* is age-appropriate for tweens and teens, and *Spiderman and Avengers* movies for older teens and adults (Common Sense Media, 2024). As children develop interest in superheroes, parents may reinforce this interest by allowing increased exposure to superhero media (Anderson et al., 2010; Coyne et al., 2017; Fraser et al., 2012), which is made easier by the availability of age-appropriate superhero content for nearly every developmental stage. With children's growing fascination with superheroes and the development of longer attention spans, we predict the amount of time spent viewing superhero media will increase across time.

#### Aggression and superheroes

Aggression in early childhood is defined as physical behaviors enacted with the intention to harm others, including hitting, kicking, biting, etc. (Liu et al., 2013). Physical aggression is fairly commonplace in early toddlerhood and peaks during the early preschool years, then gradually declines into mid- to late childhood and adolescence (Eisner and Malti, 2015). Superhero media contains high levels of aggressive acts, with more instances of aggression enacted by the hero—for the purpose of saving or helping others—than the villain (Muller et al., 2020). However, research suggests engagement with superheroes to be related to higher levels of aggressive behavior over time, and not linked with prosocial or defending behaviors (Coyne et al., 2017). While the aggressive behaviors enacted in superhero media is often portrayed as noble or justified, this distinction does not seem to translate for young children. Prosocial and defending behavior will be discussed in detail below.

The social learning theory suggests that children learn aggressive behaviors by viewing and imitating the aggressive behaviors of others they view as strong role models, particularly in situations where they identify strongly with those models (e.g., Bandura, 1969). Superheroes are powerful role models for imitation, and high identification with superheroes who behave violently may increase the likelihood of children internalizing aggressive norms. Activities including playing with superhero toys or dressing up as a superhero may give children an opportunity to integrate aggression in their sociodramatic play and superhero engagement is linked with reinforced gender stereotypes and hegemonic masculinity, which are associated with heightened aggression in boys (Coyne et al., 2022). Different forms of superhero engagement are likely distinctively related to aggressive behavior over time, with the modeling of aggressive behavior being particularly salient for learning in this age group (Tremblay, 2000).

### Prosocial and defending behavior and superheroes

Though superhero media often showcases aggressive acts, many superhero storylines center around trying to “save” or “help” bystanders, suggesting that prosocial and defending behaviors are also regularly modeled to consumers (Coyne et al., 2017). Prosocial behavior is defined as any action intended to benefit other individuals, and may include helping, sharing, or cooperation (Fraser et al., 2012) Research suggests that children begin to exhibit prosocial behaviors as early as age three (Reschke et al., 2023). Prosocial behaviors are a fundamental element of social interaction and social development in early childhood (Huber et al., 2019), and children's media, such as *Doc McStuffins* or *Daniel Tiger* focus on teaching prosocial behavior. Indeed, research suggests that viewing prosocial behavior in media is linked with increased prosocial behavior across childhood and adolescence (Coyne et al., 2018).

Defending behaviors are a type of prosocial behavior aimed at supporting disadvantaged or vulnerable victims of aggression (Salmivalli et al., 1996). These behaviors include both non-aggressive and aggressive behavior and have been observed in children as early as age three (Camodeca and Coppola, 2019). Non-aggressive defending is linked with benefits including less victimization in the school setting (Kärnä et al., 2010; Salmivalli et al., 2011) increased empathy (van Noorden et al., 2014; Lambe and Craig, 2020), and moral reasoning (Caravita et al., 2012; Thornberg and Jungert, 2013). In contrast, less is known about aggressive defending, though research indicates that individuals prone to aggression act more often as defenders than their non-aggressive peers (Huitsing and Monks, 2018).

Superheroes are portrayed as highly prosocial, using both aggressive and non-aggressive strategies to defend those in need. Children learn by observing others' actions (Bussey and Bandura, 1999), internalizing social cues to guide their own behavior (Galán et al., 2022). Superheroes represent a powerful model for shaping understanding of acceptable or unacceptable behaviors in social settings. Indeed, Coyne et al. (2017) found that superhero engagement (in general) was linked with higher levels of aggression 1 year later and was not associated with prosocial behavior or defending behaviors—aggressive or non-aggressive alike.

However, the impact of superhero engagement may depend on the type and patterns of interaction. For example, one study examining children's engagement with princess media found that it was playing pretend, rather than other types of engagement, was associated with children's body esteem and gendered-type play (Shawcroft et al., 2024). Similarly, there is likely considerable nuance in the developmental implications of superhero engagement for young children.

For example, highly identifying with superheroes and viewing high levels of superhero media early in childhood—and increasing in engagement over time—may be related to higher aggressive defending behavior, but lower prosocial behavior in general. At ages 2–3, the concept of defending is still emerging, and children may not have the capacity to understand complex storylines or characters using violence to help others. Conversely, early engagement with superhero-themed play, such as using toys or dressing up outside of the context of media, may represent typical developmental processes. These forms of sociodramatic play may be related to higher levels of prosocial behavior without promoting aggression as it focuses on creativity and role-playing.

### Gender

Research indicates gender differences in superhero engagement during early childhood, with multiple studies suggesting boys engage more with superhero media than girls (Coyne et al., 2017; Morgan et al., 2021). However, there is variation in superhero engagement, even among boys. For example, among preschool age boys, some view superhero movies or TV shows at least once a week while many others have not yet been exposed to superhero media at all (Coyne et al., 2014).

The social cognitive theory of gender development suggests that media influence is stronger when viewers relate to characters their same gender, making identification stronger (Bussey and Bandura, 1999). As most superheroes are male and exhibit traditionally masculine behaviors (Shawcroft and Coyne, 2022), boys may feel a stronger connection and identification with superheroes than girls (Baker and Raney, 2007). Indeed, effects of superhero engagement tend to be stronger for boys than girls (Coyne et al., 2021). Additionally, gender differences in social behaviors may impact superhero identification. Among preschool-aged children, boys are more likely to exhibit aggression than girls (Card et al., 2008), and girls more often engage in prosocial and non-aggressive defending behaviors than boys (Padilla-Walker and Carlo, 2014), especially later in development (Jambon et al., 2019). The gender-linked model of aggression (Ostrov et al., 2014) supports this idea in that socialization processes reinforce gender differences and reinforce gender-typical behaviors. Superhero engagement likely plays a role in this socialization by amplifying gendered patterns of aggression, particularly for young boys who highly identify with superhero characters.

## Current study

The purpose of this study is to examine the growth of superhero engagement across early childhood (between the ages of 2.5 and 6.5 years old). We examined predictors of the growth of superhero engagement over childhood and longitudinal outcomes of superhero engagement, specifically examining aggression, prosocial, and defending behaviors. The study aims and hypotheses are the following:

*RQ1: What are the intraindividual trajectories of superhero engagement across early childhood?*

H1a: We predict that levels of superhero identification and superhero media will increase over time.

H1b: We predict the levels of playing with superhero toys and dressing up as superheroes will decrease over time.

H1c: We expect there to be variation in these trajectories depending on individual children.

*RQ 2: What predicts individual differences in intraindividual superhero engagement trajectories?*

H2: We hypothesize that higher levels of child aggression, being male, higher television time, and lower parent education will predict higher, increasing, and earlier levels of superhero engagement over time.

*Aim 3: Does the growth of superhero engagement predict child outcomes such as aggression, prosocial, and defending behaviors over time?*

H3: For children that start high in their superhero engagement and increase rapidly will predict higher levels of aggression, lower levels of prosocial behavior, higher levels of aggressive defending behaviors, and lower levels of non-aggressive defending behaviors.

## Methods

### Participants

Participants for this study were taken from Project M.E.D.I.A., an ongoing longitudinal study examining the effects of a media-saturated world on child development. In 2017, 500 primary caregivers (Female = 484, Male = 10, missing = 6) were originally recruited to participate in this study along with their children who were under a year old at the time. Some participants were found through the Colorado Office of Health and Vital records, which helped the study, identify residents who had a child within the last year of when the study was beginning. Participants were also recruited using flyers in pediatrician offices, free clinics, social services offices, businesses focused on entertainment for young children, public parks and play spaces, and referrals from friends who participated in the study.

The analytical sample was taken from Waves 3–7 of this longitudinal project, when we began to examine superhero engagement. This included 430 children (*M* age = 29.17 months; SD = 3.77 months; 42% male) and their parents. See Table 1 for additional demographic details. The retention rate for these waves was 76%. For simplicity, we will refer to these as Waves 1–5 throughout. Families that had stopped participating in the study overall and were missing data at all five time points for all variables were not included in the analysis. A Little's MCAR missing data analysis was conducted and the data were missing at random (*p* > 0.05).

**Table 1:** Demographic statistics.

**Variable**	**%**
**Caregiver marital status**	
Married	66.30
Single-never married	8.70
Unmarried- living with partner	9.0
Divorced, Separated, or Widowed	2.80
**Caregiver education**	
High school (or equivalent) or less	11.00
Some college or vocational degree	26.70
Bachelor's degree	30.00
Graduate degree	19.30
**Combined household income**	
Less than $20,000	8.30
Between $20,000 and $50,000	21.80
Between $50,000 and $80,000	27.20
Between $80,000 and $100,000	12.50
Above $100,000	30.20
**Public assistance**	
Currently (at the time of data collection)	25.90
In the past year, but not currently	5.00
In the past, but not in the last year	18.80
Have never received public assistance	50.20
**Ethnicity**	
Latinx	21.00
Not latinx	79.00
**Race**	
White	67.00
Black	6.30
Asian American	2.40
American Indian/Alaskan Native	0.70
Multi-racial	4.20
Other	19.30

### Procedure

After giving informed consent, parents completed a series of online questionnaires examining their child's superhero engagement, aggression, prosocial behavior, and defending behaviors once a year for a total of 5 years.

### Measures

#### Superhero engagement (Waves 1–5)

Parents reported on four aspects of their child's engagement with superheroes: identification with superheroes, toys, pretend play, and media (each was measured with one question; Coyne et al., 2016). For identification, parents were shown 17 images of highly popular superheroes and were asked to choose the superhero their child identified with the most. Parents could also choose a superhero not on the list. They were then asked how much their child *identified* with this identified superhero on a 7-point Likert-type scale (1 = not at all to 7 = highly identifies with). For toys, parents were asked how frequently their child plays with *toys* relating to any superhero. This measure was rated on a 7-point Likert-type scale (1 = less than once a month to 7 = 6 or more times per week). For pretend play, parents were asked how frequently their child *dresses up* like a superhero for pretend play. This measure was rated on a 7-point Likert-type scale (1 = less than once a month to 7 = 6 or more times per week). For *media*, parents reported how frequently their child viewed television shows or movies (including DVDs) portraying superheroes, also measured on a 7-point Likert-type scale (1 = never to 7 = 2–3 times per week).

#### Aggression (Wave 1 and 5)

Child aggression at Wave 1 was measured using the Child Behavior Checklist 1 ½−5 (CBCL 1 ½−5; Achenbach and Rescorla, 2000). This 100-item measure provides eight distinct subscales, including one on aggressive behavior that consists of 18 items. Example items include, “gets in many fights,” “hits others,” and “physically attacks others.” Parents responded on a three-point Likert-type scale from 0 *(Not true, as far as you know)* and 2 *(Very true or often true)* for each item. Items in each scale are averaged and scores are normed in comparison to nationally representative datasets of children the same age (Achenbach and Rescorla, 2000). Higher scores indicate higher levels of aggression. Reliability was acceptable (α = 0.87).

For Wave 5, the CBCL 6–18 scale was used, which is more appropriate for children of older ages. Again, the aggression scale consisted of 18 items measured on the same scale. Example items included, “gets in many fights,” “physically attacks people,” “threatens people.” Reliability was also acceptable at this wave (α = 0.87).

#### Prosocial behavior (Wave 5)

Children's prosocial behavior was measured using five items from the Preschool Social Behavior Survey adapted for parental reporters (PSBS; Crick et al., 1997). In this measure parents reported on a five-point Likert type scale from 1 (*Never or almost never true*) to 5 (*Always or almost always true*) about their child's prosocial behavior in the peer group. Higher scores on these items indicated higher levels of prosocial behavior. Example items included “Your child says or does nice things for other kids” and “Your child is good at sharing and taking turns.” These items were averaged together and had a Cronbach's alpha of α = 0.79.

#### Aggressive defending behaviors

Aggressive defending contained three items also from the PSBS (Crick et al., 1997), measured on the same scale as above. Higher scores corresponded to higher levels of aggressive defending behaviors. Example items included “Your child aggressively defends those who are being physically bullied by other classmates. For example, he/she will not hesitate to push or hit the bully to put an end to the bullying.” Cronbach's alpha was found to moderate (α = 0.68).

#### Non-aggressive defending behaviors

Non-aggressive defending was also measured using contained three items also from the PSBS (Crick et al., 1997), also measured on the same scale as above. An example is: “Your child assertively, but not aggressively, defends those who are being physically bullied by other classmates. For example, he/she may tell the bully to stop.” Higher scores indicated higher levels of non-aggressive defending in the child. These items were averaged together and had an acceptable Cronbach's alpha of α = 0.78.

### Analysis plan

To examine superhero engagement over time, we ran four growth curve models (identification, play, dress, and media). If the variance was significant in each model, we conducted an additional growth mixture model to explore differential trajectories. To determine the number of classes, we examined two information criteria: the Bayesian Information Criterion (BIC) and the sample size-adjusted BIC (SABIC). Given that the BIC and SABIC may indicate differing numbers of classes, we also employed the LL-Diff (Log Likelihood Difference) test, examined class sizes, and took entropy into account (a measure of how well cases classify).

Next, the three-step approach in Mplus (v 8.8) was used to determine predictors of the change patterns of superhero engagement and how outcome variables differ across the patterns.

Predictors included biological sex, aggression, parental education, and overall television time (all at Wave 1). Outcomes included aggression, prosocial behavior, aggressive defending, and non-aggressive defending (all at Wave 5).

## Results

Bivariate correlations were run for all variables. In general, the four types of superhero engagement were positively and moderately correlated across all waves. There were weak associations with child behaviors depending on the wave. Table 2 provides the correlations for the final wave only for parsimony (though full results can be provided by contacting the primary author). Additionally, a multivariate analysis of variance was used to examine sex differences for all major variables (Wave 5 is shown for parsimony). The analysis revealed a significant multivariate effect, *F*_(8.267)_ = 8.23, *p* < 0.001, η^2^ = 0.20 (see Table 3 for all results). Boys were higher than girls on all superhero engagement. Additionally, girls were higher than boys on prosocial behavior and non-aggressive defending.

**Table 2:** Bivariate correlations for main variables.

**Variables**	**1**	**2**	**3**	**4**	**5**	**6**	**7**	**8**
1. Superhero identification	–							
2. Superhero toys	0.608^**^	–						
3. Superhero media	0.466^**^	0.644^**^	–					
4. Superhero dress	0.548^**^	0.688^**^	0.453^**^	–				
5. Aggression	0.113^*^	0.094	0.170^**^	0.076	–			
6. Prosocial behavior	−0.070	−0.105	−0.078	−0.060	−0.298^**^	–		
7. Aggressive defending	0.025	−0.009	−0.021	0.069	0.181^**^	−0.087	–	
8. Non-aggressive defending	0.031	0.033	0.026	0.079	−0.119^*^	0.464^**^	0.244^**^	–

^**^Correlation is significant at the 0.01 level (2-tailed); ^*^Correlation is significant at the 0.05 level (2-tailed).

Data is only shown for the final wave for parsimony.

**Table 3:** Sex differences for major variables.

**Variables**	**Boys**		**Girls**		**F-value**	**Effect size**
	**M**	**SD**	**M**	**SD**		
Superhero identification	3.92	1.97	2.71	1.71	28.61^***^	0.10
Superhero toys	3.97	1.86	2.59	1.59	41.74^***^	0.13
Superhero dress	2.70	1.88	2.13	1.36	9.82^**^	0.04
Superhero media	4.81	1.81	4.06	1.86	11.38^***^	0.04
Aggression	1.25	0.24	1.19	0.23	3.44	0.01
Prosocial behavior	4.20	0.50	4.39	0.52	9.52^**^	0.03
Aggressive defending	1.61	0.63	1.53	0.70	0.84	0.001
Non-aggressive defending	2.97	0.86	3.23	0.86	6.30^*^	0.02

^*^p <0.05; ^**^p <0.01; ^***^p <0.001.

**Figure 1:**
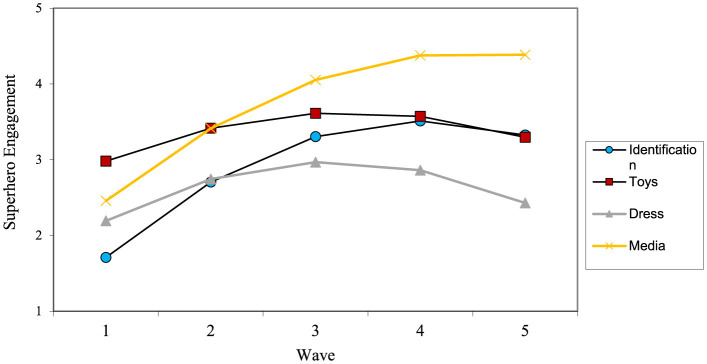
Superhero engagement over time.

Next, a series of growth curve analyses were conducted for all four types of superhero engagement. The results of all growth curve analyses can be found in Table 4 and a model of the growth of superhero engagement, play, dressing up, and media can be found in Figure 1.

**Table 4:** Growth curve analysis for all types of superhero engagement.

**Type of superhero engagement**	**Overall results**			**Variance**		
	**Intercept**	**Slope**	**Quadratic**	**Intercept**	**Slope**	**Quadratic**
Superhero identification	1.71^***^	1.19^***^	−0.20^***^	3.60^***^	1.34^**^	0.05^*^
Superhero toys	2.98^***^	0.55^***^	−0.12^***^	3.93^***^	1.53^***^	0.07^***^
Superhero dress	2.19^***^	0.717^***^	−0.16^***^	2.22^***^	1.13^***^	0.05^***^
Superhero media	2.46^***^	1.11^***^	−0.16^***^	3.47^***^	0.88^**^	0.03^*^

^*^p < 0.05; ^**^p < 0.01; ^***^p < 0.001.

### Superhero identification

**Figure 2:**
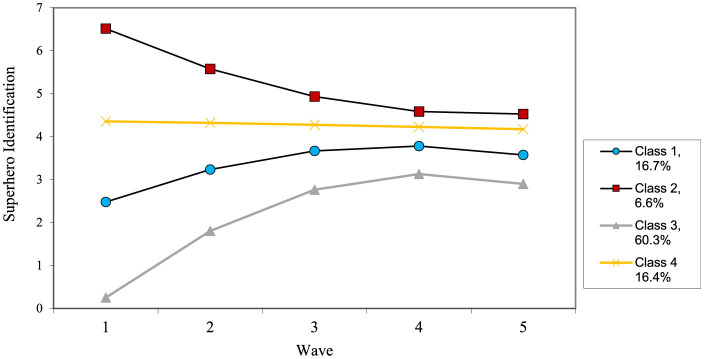
Estimated change patterns for superhero identification.

The growth curve for superhero identification was acceptable, χ^2^_(36)_ = 140.94, *p* < 0.001, CFI = 0.86, TLI = 0.86, RMSEA = 0.08, and revealed a significant intercept, slope, and quadratic term. In general, children grew in their identification with superheroes, which leveled out toward the end of early childhood. There was also significant variance in all three, so we conducted a growth mixture model to explore different trajectories (see Figure 2 for final trajectories).

Table 5 shows the fit improvement when exploring each class. An examination of the fit statistics revealed that a four-class solution fit the model best for superhero identification. Class 1 (16.7%: moderate growth) started out relatively low but grew over time, stabilizing by the later waves. Class 2 (6.6%: high decreasers) started out with very high levels of superhero identification that dropped over time. This group had the highest superhero identification at the final wave. Class 3 (60.3%: low increasers) represented the majority of children, who started out very low on superhero identification, but then gradually increased over time, stabilizing toward the end of early childhood. Class 4 (16.4%: moderate stable) started with moderate levels of superhero identification that did not increase over the course of early childhood.

**Table 5:** Relative model fit by number of classes for superhero identification.

**Classes**	***n***	**Log-likelihood**	**Entropy**	**BIC**	**SABIC**	**LLR test**
1	282	−3,484.86	—	–	1,583.34	—
2	350,151	−3,484.86	0.81	7,081.62	7,024.49	*p* < 0.001
3	51,96,354	−3,439.19	0.82	7,015.15	6,945.32	*p* < 0.001
**4**	**72,27,324,78**	**−3,388.66**	**0.88**	**6,938.94**	**6,856.42**	***p*** **<** **0.001**
5	77,72,30,274,48	−3,380.14	0.81	6,946.77	6,851.55	*p <* 0.001

Bolded line indicates best fitting model.

In terms of predictors, boys were more likely to be in the high decreasers and moderate stable groups (all comparisons *p* < 0.05). Additionally, low increasers viewed significantly less television than all the other three groups (all comparisons *p* < 0.05). For outcomes, high decreasers showed the highest levels of aggressive defending compared to all other groups (*p* < 0.001).

### Superhero toys

The growth curve for superhero toys showed adequate fit, Model fit was moderate, χ^2^_(6)_ = 7.22, *p* = 0.30, CFI = 0.99, TLI = 0.99, RMSEA = 0.02 and revealed a significant intercept, slope and quadratic terms, along with significant variance in each result. In general, playing with superhero toys is moderate around the first wave, peaks around wave 3, and then decreases by the final wave.

**Figure 3:**
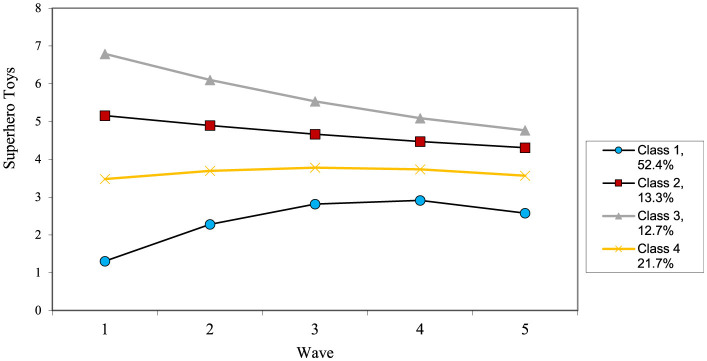
Estimated change patterns for superhero toys.

The growth mixture model suggested that a four-class solution fit the model best (see Table 6 for results and Figure 3 for final trajectories). The pattern was similar to superhero identification with a high decreasing group (12.7%), moderate decreasing (13.3%), moderate stable (21.7%), and low increasing curve (52.4%). For predictors, parent education was higher in the low increasers group compared to other groups. The high decreasers group was more likely to consist of boys as compared to the moderate stable and low increasers. However, the moderate group did not differ in sex compared to any group. Finally, the high increasers had higher levels of television than the low increasers and moderate stable group. For outcomes, aggressive defending was significantly higher in the high decreasers and moderate group as compared to the moderate decreasers and low increasers. Additionally, the high decreasers group had significantly higher levels of aggression than all other groups, despite aggression not being different between these groups at the initial wave. All comparisons described above were significant (*p* < 0.05).

**Table 6:** Relative model fit by number of classes for superhero toys.

**Classes**	***n***	**Log-likelihood**	**Entropy**	**BIC**	**SABIC**	**LLR test**
1	500	−3,370.78	—	6,828.57	6,784.13	—
2	319,181	−3,299.76	0.78	6,711.37	6,654.24	*p* < 0.001
3	268,153,78	−3,254.46	0.82	6,645.65	6,575.82	*p* < 0.001
**4**	**275,53,75,97**	**−3,215.53**	**0.85**	**6,592.63**	**6,510.11**	***p*** **<** **0.001**
5	83,257,48,12,100	−3,210.86	0.81	6,608.17	6,512.94	*p <* 0.001

Bolded line indicates best fitting model.

### Superhero dress

**Figure 4:**
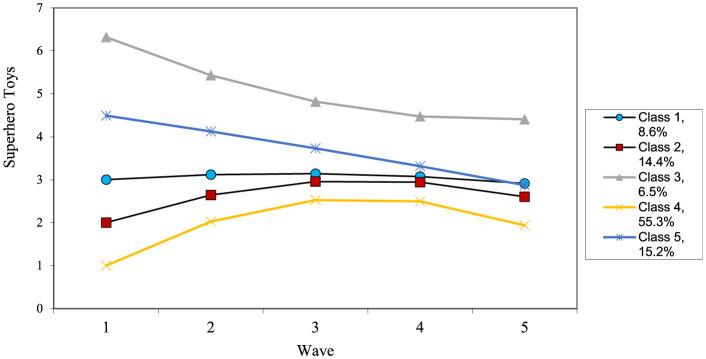
Estimated change patterns for superhero dress.

The growth curve for superhero dress showed adequate fit, χ^2^_(6)_ = 23.57, *p* < 0.001, CFI = 0.96, TLI = 0.93, RMSEA = 0.07 and revealed a significant intercept, slope and quadratic terms, along with significant variance in each. The growth mixture model revealed a five-class solution (see Table 7 for results and Figure 4 for final trajectories). There was a high decreaser (6.5%), moderate decreaser (15.2%), moderate (8.6%), low increaser (14.4%), and lowest increaser group (55.3%). Television viewing was higher in the high decreaser and moderate decreaser groups as compared to the lower groups. Additionally, the moderate decreasing group had higher initial levels than the lowest group. The low increaser group was also more likely to include boys than the moderate or lowest increasing groups. In terms of outcomes, the low increaser group had significantly higher levels of aggression than all other groups apart from the moderate decreasing group (all comparisons *p* < 0.05).

**Table 7:** Relative model fit by number of classes for superhero dress.

**Classes**	***n***	**Log-likelihood**	**Entropy**	**BIC**	**SABIC**	**LLR test**
1	500	−3,210.88	—	6,508.79	6,464.35	—
2	99, 402	−3,084.55	0.87	6,280.99	6,223.86	*p* < 0.001
3	66,70,365	−3,019.95	0.89	6,176.67	6,106.84	*p* < 0.001
4	31,34,365,71	−2,990.31	0.89	6,142.24	6,509.72	*p* < 0.001
**5**	**37,62,36,295,71**	**−2,892.79**	**0.90**	**5,845.60**	**5,876.87**	***p** **<*** **0.001**
6	326,38,26,45,43,23	−2,957.50	0.85	6,126.36	6,018.44	*p <* 0.001

Bolded line indicates best fitting model.

### Superhero media

**Figure 5:**
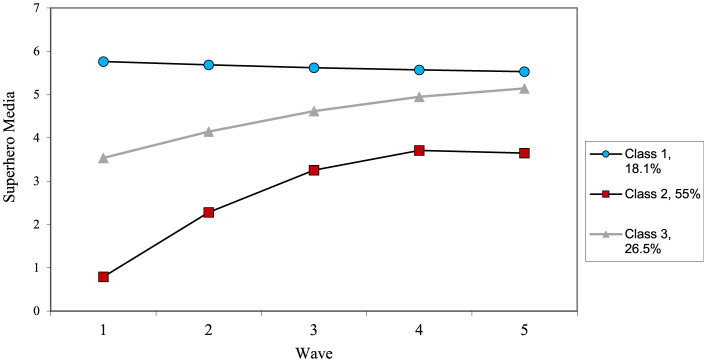
Estimated change patterns for superhero media.

The growth curve for superhero media also showed adequate fit, χ^2^_(6)_ = 35.35, *p* < 0.001, CFI = 0.95, TLI = 0.92, RMSEA = 0.09, and revealed a significant intercept, slope and quadratic terms, along with significant variance in each. In general, superhero media increased dramatically before leveling off at the final wave. The growth mixture model revealed a three-class solution (see Table 8 for results and Figure 5 for final trajectories). There was a high stable group (18.8%), a moderate increasing group (26.2%), and a low increasing group (55.0%).

**Table 8:** Relative model fit by number of classes for superhero media.

**Classes**	***n***	**Log-likelihood**	**Entropy**	**BIC**	**SABIC**	**LLR test**
1	500	−3,434.28	—	6,955.60	6,911.16	—
2	177,324	−3,371.86	0.77	6,855.62	6,798.40	*p* < 0.001
**3**	**94,289,118**	**−3,324.63**	**0.78**	**6,821.78**	**6,751.96**	***p*** **<** **0.001**
4	194,122,102,83	−3,324.63	0.71	6,810.89	6,728.37	*p* < 0.001

Bolded line indicates best fitting model.

For predictors, the high stable groups were more likely to be male, view more television, have lower levels of parental education, and higher levels of aggression at the initial time point compared to the low increasing group. Additionally, this group was more likely to be male than the moderate group. Finally, the low increasing group tended to view less television in general than the moderate group. For outcomes, the moderate increasing group had higher levels of aggression and aggressive defending behavior than both the high stable and low increasing group. Additionally, the high stable group had higher levels of non-aggressive defending behavior than the high increasing group. All comparisons are significant at *p* < 0.05.

## Discussion

This study examined trajectories of growth in four dimensions of superhero engagement across early childhood, including superhero identification, play with superhero toys, superhero dress-up, and viewing of superhero media. Our model explored child aggression, gender, TV time, and parent education as predictors of individual superhero growth trajectories. Further, we explored superhero engagement growth trajectories as predictors of aggression, prosocial behavior, and defending behaviors over time. Results showed mixed support for our hypotheses. Each type of superhero engagement (identification, toys, dress up, media) had a different average pattern of change, with considerable variability in individual trajectories across the 5 years of data collection.

### Growth in domains of superhero engagement

The first aim was to understand different trajectories of superhero engagement across early childhood. Though some research has found that general superhero engagement tends to be somewhat stable across 1 year in early childhood (Coyne et al., 2014), no research has examined trajectories of different types of engagement.

In general, most types of superhero engagement were low to moderate for children around 2 years of age, with playing with superhero toys beginning at higher level compared to other types of engagement. Then, superhero engagement of all types increased after age 2. These findings align with previous research suggesting that play evolves across development, with imaginative play a prominent role in preschool-age children (Fisher et al., 2008), transitioning to socially interactive play including rules and role-based activities in middle childhood (Fromberg and Bergen, 2012).

Superhero identification and viewing superhero media tended to increase dramatically from ages 2–5, before leveling off around age 6 (Hypothesis 1a). Conversely, playing with superhero toys and dressing up as superheroes tended to increase more slowly, peaking around age 4 before declining over the next 2 years, somewhat confirming Hypothesis 1b. This peak in superhero identification around age four confirms previous findings that children's identification with media characters is often at its highest during this developmental stage (Hoffner, 1996). Overall, our findings show that superhero engagement across early childhood is not static. It changes significantly, with certain types of engagement being more prominent at different ages.

### Superhero engagement growth trajectories

We identified a range of intraindividual growth trajectories across each domain of superhero engagement (Hypothesis 1c), suggesting that children's engagement changes in diverse ways over time. Unique patterns emerged within each of the four domains, highlighting distinctions between them. Generally, superhero identification, toys, and dress-up each had a minority group that began with very high engagement and declined over time, though the size of these groups and the steepness of the declines varied. For superhero dress-up, one group began with moderately high engagement at age two and remained more engaged than peers at age five despite declines. Each domain also included a moderate stable group and a low increasing group, with variations in group size and trajectory steepness. These patterns align with research showing that children's play adapts to community, cultural, gender, socioeconomic, and family factors (Guest, 2013; Sandberg and Meyer-Bahlburg, 1994).

For superhero media, only three trajectories emerged, fewer than in other domains. This was the only type to feature a very high stable group, maintaining high viewing from age 2–6. The other two trajectories were a moderate increasing group and a very low increasing group. These patterns are consistent with prior research showing distinct, stable subgroups in children's media use (Vandewater and Lee, 2009).

### Predictors of superhero engagement

To answer research question two, we examined other factors that might predict the different trajectories we identified. In general, television viewing at age two was the strongest predictor of initially high or moderate superhero engagement over time, though this tended to decrease in strength for most types of engagement (apart from media). For gender, boys were more likely to have higher and moderate trajectories of superhero engagement than girls, for all domains other than superhero dress-up. This confirms existing research that boys have higher average levels of superhero engagement than girls (Coyne et al., 2014). Higher parent education was related to lower levels of superhero toy play and watching superhero media. This is not surprising, considering higher educated parents may be more wary of media in general (Rideout and Robb, 2020), and may associate both superhero toys and media with aggression, perhaps limiting these types of superhero engagement for their children. Additionally, child aggression at age two only predicted the high stable media group, meaning that children who exhibit aggressive behavior as toddlers tend to view much higher levels of superhero media than other children, at age two and continuing across early childhood. Overall, these findings largely support Hypothesis 2.

### Outcomes of superhero engagement

Finally, research question three explored outcomes associated with different superhero engagement trajectories. Children in the high decreasing groups for superhero identification and toy play showed higher levels of aggressive defending and overall aggression compared to other groups. These findings partially confirm Hypothesis 3 and align with key principles of social learning theory (Bandura, 1969). Superheroes represent strong, powerful, attractive role models (Shawcroft and Coyne, 2022). Identifying with superheroes early on and then reinforcing this by playing with superhero-themed toys, may prime a child to engage in higher levels of aggressive defending later in life. Indeed, superhero behavior can best be characterized as aggressive defending, in which a character frequently uses aggression against villains to save others. Similarly, children who identify with superheroes might model similar aggressive defending behaviors toward their peers or family members, in the present study, aggressive defending was the only social outcome related to any trajectory for superhero identification.

There were two notable findings for superhero media. First, children with moderate increasing media use showed higher levels of aggression than both the low increasing and high stable groups. This partially supports our hypothesis but raises questions about why aggression was higher in this group compared to the high stable group. Previous research has linked early superhero media exposure to later aggression (Coyne et al., 2014, 2017). However, we also found that the high stable group displayed the highest levels of non-aggressive defending behavior, contradicting prior findings. This group also exhibited general risk factors—high television use, being male, low parental education, and initially high aggression—which may explain some of the patterns observed. It is possible that their aggression decreased over time, a common trend in early childhood (Tremblay, 2000), or that early exposure to defending themes fostered increases in non-aggressive defending. Alternatively, a sharp rise in superhero media consumption after a moderate start may be linked to negative behavioral outcomes across early childhood. Additionally, we did not measure superhero *content*, which may also account for some of the results. Superhero content for young children is often more educational and prosocial in nature (e.g., *PJ Masks, Paw Patrol, SuperWhy*). It is possible that our high stable group started out watching this type of content, whereas the moderate group viewed content that was less developmentally appropriate. This highlights the importance of assessing content when studying media effects. We suggest that future research directly assess media content to explore greater insights and understanding about the effects of media use on child outcomes.

A final unexpected finding was that, for the domain of superhero dress-up, children in the low increasing group were most likely to be aggressive out of all five groups. This was somewhat surprising, as it represented a departure from the types of patterns we saw in the domains of superhero identification and toy play. This low increasing group followed a very similar pattern to our lowest (and majority) group for dress-up, but was just slightly higher and more likely to be male. It is possible that children with low initial levels of dress-up engage highly in other types of superhero engagement (i.e., media, identification, or toy play). Given that children in this group were much more likely to be boys, they may have associated dressing up as a feminine activity and chose instead to engage with superheroes in other ways. The social cognitive theory of gender (Bussey and Bandura, 1999) supports this premise, suggesting that boys may be prescribing to masculine norms when engaging with superheroes. Apart from this finding, superhero dress-up was the domain of superhero engagement least related to social outcomes for children.

### Implications, limitations, and future research

There are several implications of the current study for parents, researchers, educators, and producers of superhero merchandise and media. Notably, there are many different types of superhero engagement, and not all children use them in the same way. Thus, researchers should continue to examine different types of superhero engagement when thinking about longer term effects on children. Additionally, high superhero identification and play with superhero toys in very early childhood appears to be most risky for the development of aggressive defending and aggression (for toys only)—even if it decreases over time. Parents may wish to avoid buying superhero toys for their children at very young ages, which may prompt higher levels of identification. Educators may wish to discuss superhero engagement with their classes. Anecdotally, many educators use superheroes as role models to promote positive behavior within their classrooms, and research has outlined the utility of using superheroes in therapy setting with children (Rubin and Livesay, 2006). However, not all superhero engagement was related to positive outcomes—and no superhero engagement was related to prosocial behavior. Instead, educators may wish to highlight any prosocial or non-aggressive defending that superheroes engage in, or perhaps focus on other role models that are less controversial in nature. Additionally, producers of superhero content may wish to create content that models high levels of non-aggressive defending, particularly when it might be viewed among young children.

Though this study had many strengths including longitudinal data collected over 5 years and multiple types of superhero engagement, there were some notable limitations. First, we did not measure the content of superhero programs. Clearly, some programs are more violent than others and we hope that future research examines how engagements with specific superheroes relate to different childhood outcomes. Additionally, all the data was parent report, which may result in some shared method variance. Future research might use child-report or observational studies to better understand superhero engagement in young children. Additionally, the sample tended to be predominantly White. Most superheroes are also White, perhaps increasing identification among children, but future research should test these results in more diverse samples. Finally, superhero engagement is likely to change more rapidly than just from year to year, as measured by our study. Thus, future research could examine the growth of superhero engagement in shorter time periods (such as from month to month).

Overall, this study offers important academic and societal contributions to the study of superheroes. By distinguishing among identification, media viewing, toy play, and dress-up, we demonstrate that superhero engagement is not a uniform construct; different modes of engagement follow distinct developmental paths and predict different social outcomes. This advances media effects theories and the superhero research literature by emphasizing the role of engagement type in shaping behavior. On a societal level these findings inform strategies for parents, educators, and media producers. Parents can guide children toward healthier modes of engagement, educators can emphasize prosocial lessons from superhero narratives, and media creators can design content that promotes non-aggressive defending. Recognizing the multidimensional nature of superhero engagement over time allows for more targeted approaches to supporting positive child development.

## Data Availability

The raw data supporting the conclusions of this article will be made available by the authors, without undue reservation.

## References

[B1] Achenbach T. M. Rescorla L. A. (2000). Manual for the ASEBA Preschool Forms and Profiles. Burlington, VT: University of Vermont, Research Center for Children, Youth, and Families.

[B2] Anderson C. A. Shibuya A. Ihori N. Swing E. L. Bushman B. J. Sakamoto A.. (2010). Violent video game effects on aggression, empathy, and prosocial behavior in Eastern and Western countries: a meta-analytic review. Psychological Bulletin 136, 151–173. 10.1037/a0018251 20192553

[B3] Baker K. Raney A. A. (2007). Equally super?: gender-role stereotyping of superheroes in children's animated programs. Mass Commun. Soc. 10, 25–41. 10.1080/15205430709337003

[B4] Bandura A. (1969). “Social learning theory of identificatory processes,” in Handbook of Socialization Theory and Research, ed. D. A. Goslin (Chicago: Rand McNally), 213–262.

[B5] Bauer M. Georgeson A. McNamara C. Wakefield B. H. King T. S. Olympia R. P. (2017). Positive and negative themes found in superhero films. Clin. Pediatr. 56, 1293–1300. 10.1177/0009922816682744 28006958

[B6] Bussey K. Bandura A. (1999). Social cognitive theory of gender development and differentiation. Psychol. Rev. 106, 676–713. 10.1037/0033-295X.106.4.676 10560326

[B7] Camodeca M. Coppola G. (2019). Participant roles in preschool bullying: the impact of emotion regulation, social preference, and quality of the teacher-child relationship. Soc. Dev. 28, 3–21. 10.1111/sode.12320

[B8] Caravita S. C. S. Gini G. Pozzoli T. (2012). Main and moderated effects of moral cognition and status on bullying and defending. Aggress. Behav. 38, 456–468. 10.1002/ab.21447 22898969

[B9] Card N. A. Stucky B. D. Sawalani G. M. Little T. D. (2008). Direct and indirect aggression during childhood and adolescence: a meta-analytic review of gender differences, intercorrelations, and relations to maladjustment. Child Dev. 79, 1185–1229. 10.1111/j.1467-8624.2008.01184.x 18826521

[B10] Cohen J. (2001). Defining identification: a theoretical look at the identification of audiences with media characters. Mass Commun. Soc. 4, 245–264. 10.1207/S15327825MCS0403_01

[B11] Common Sense Media (2024). Available online at: https://www.commonsensemedia.org

[B12] Coyne S. M. Linder J. R. Rasmussen E. E. Nelson D. A. Birkbeck V. (2016). Pretty as a princess: longitudinal effects of engagement with Disney princesses on gender stereotypes, body esteem, and prosocial behavior in children. Child Dev. 87, 1909–1925. 10.1111/cdev.12569 27315773

[B13] Coyne S. M. Linder J. R. Rasmussen E. E. Nelson D. A. Collier K. M. (2014). It's a bird! It's a plane! It's a gender stereotype!: Longitudinal associations between superhero viewing and gender-stereotyped play. Sex Roles 70, 416–430. 10.1007/s11199-014-0374-8

[B14] Coyne S. M. Padilla-Walker L. M. Holmgren H. G. Davis E. J. Collier K. M. Memmott-Elison M. K.. (2018). A meta-analysis of prosocial media on prosocial behavior, aggression, and empathic concern: a multidimensional approach. Dev. Psychol. 54, 331–347. 10.1037/dev0000412 29083208

[B15] Coyne S. M. Rogers A. A. Shawcroft J. Hurst J. L. (2021). Dressing up with disney and make-believe with marvel: the impact of gendered costumes on gender typing, prosocial behavior, and perseverance during early childhood. Sex Roles 85, 301–312. 10.1007/s11199-020-01217-y

[B16] Coyne S. M. Shawcroft J. Linder J. R. Graver H. Siufanua M. Holmgren H. (2022). Making men of steel: superhero exposure and the development of hegemonic masculinity in children. Sex Roles 86, 1–15. 10.1007/s11199-022-01293-2

[B17] Coyne S. M. Stockdale L. Linder J. R. Nelson D. A. Collier K. M. Essig L. W. (2017). Pow! Boom! Kablam!: effects of viewing superhero programs on aggressive, prosocial, and defending behaviors in preschool children. J. Abnorm. Child Psychol. 45, 1523–1535. 10.1007/s10802-016-0253-6 28070754

[B18] Crick N. R. Casas J. F. Mosher M. (1997). Relational and overt aggression in preschool. Dev. Psychol. 33, 579–588. 10.1037/0012-1649.33.4.579 9232373

[B19] Dinella L. (2017). Halloween costume choices: reflections of gender development in early childhood. J. Genet. Psychol. 178, 165–178. 10.1080/00221325.2017.1295223 28402183

[B20] Dinham J. Chalk B. (2018). It's Arts Play: Young Children Belonging, Being, and Becoming Through the Arts. Oxford: Oxford University Press.

[B21] Eisner M. Malti T. (2015). “Aggressive and violent behavior,” in Handbook of Child Psychology and Developmental Science: Socioemotional Processes, eds. R. M. Lerner and M. Lamb, Vol. 3, 7th Edn. (Hoboken, NJ: Wiley-Blackwell), 794–841.

[B22] Fisher K. R. Hirsh-Pasek K. Golinkoff R. M. Gryfe S. G. (2008). Conceptual split? Parents' and experts' perceptions of play in the 21st century. J. Appl. Dev. Psychol. 29, 305–316. 10.1016/j.appdev.2008.04.006

[B23] Fraser A. M. Padilla-Walker L. M. Coyne S. M. Nelson L. J. Stockdale L. A. (2012). Associations between violent video gaming, empathic concern, and prosocial behavior toward strangers, friends, and family members. J. Youth Adolesc. 41, 636–649. 10.1007/s10964-012-9742-2 22302216

[B24] Fromberg D. P. Bergen D. (2012). Play From Birth to Twelve: Contexts, Perspectives, and Meanings. Routledge.

[B25] Galán C. A. Feldman J. S. McClaine R. N. (2022). Using the social information processing model to understand gender differences in the manifestation and frequency of aggression. Aggress. Violent Behav. 66:101766. 10.1016/j.avb.2022.101766

[B26] Guest A. M. (2013). Cultures of play during middle childhood: interpretive perspectives from two distinct marginalized communities. Sport Educ. Soc. 18, 167–183. 10.1080/13573322.2011.555478

[B27] Harriger J. A. Wick M. R. Mendez K. Barnett B. (2022). With great power comes great responsibility: a content analysis of masculinity themes in superhero movies. Psychol. Men Masculin. 23, 353–364. 10.1037/men0000398

[B28] Hoffner C. (1996). Children's wishful identification and parasocial interaction with favorite television characters. J. Broadcast. Electron. Media 40, 389–402.

[B29] Huber L. Plötner M. Schmitz J. (2019). Social competence and psychopathology in early childhood: a systematic review. Eur. Child Adolesc. Psychiatr. 28, 443–459. 10.1007/s00787-018-1152-x 29637284

[B30] Huesmann L. R. Lagerspetz K. Eron L. D. (1984). Intervening variables in the TV violence-aggression relation: evidence from two countries. Dev. Psychol. 20, 746–775. 10.1037/0012-1649.20.5.746

[B31] Huitsing G. Monks C. P. (2018). Who victimizes whom and who defends whom? A multivariate social network analysis of victimization, aggression, and defending in early childhood. Aggress. Behav. 44, 394–405. 10.1002/ab.21760 29577329 PMC6033031

[B32] Jambon M. Madigan S. Plamondon A. Jenkins J. (2019). Developmental trajectories of physical aggression and prosocial behavior in early childhood: family antecedents and psychological correlates. Dev. Psychol. 55, 1211–1225. 10.1037/dev0000714 30945885

[B33] Kärnä A. Voeten M. Poskiparta E. Salmivalli C. (2010). Vulnerable children in varying classroom contexts: bystanders' behaviors moderate the effects of risk factors on victimization. Merrill-Palmer Quart. 56, 261–282. 10.1353/mpq.0.0052

[B34] Lambe L. J. Craig W. M. (2020). Peer defending as a multidimensional behavior: development and validation of the Defending Behaviors Scale. J. Sch. Psychol. 78, 38–53. 10.1016/j.jsp.2019.12.001 32178810

[B35] Liu J. Lewis G. Evans L. (2013). Understanding aggressive behavior across the lifespan. J. Psychiatr. Ment. Health Nurs. 20, 156–168. 10.1111/j.1365-2850.2012.01902.x 22471771 PMC3411865

[B36] Maccoby E. E. Wilson W. C. (1957). Identification and observational learning from films. J. Abnorm. Soc. Psychol. 55, 76–87. 10.1037/h0043015 13462664

[B37] Martin J. (2023). Superhero media as a potential context for investigating children's understanding of morally relevant events. Liberi et Liberi 12, 11–35. 10.21066/carcl.libri.12.1.1

[B38] Morgan C. H. Morrongiello B. A. Schwebel D. C. (2021). Short- and long-term effects of superhero media on young children's risk-taking behaviors. J. Pediatr. Psychol. 46, 779–789. 10.1093/jpepsy/jsaa133 33982100

[B39] Morgan C. H. Schwebel D. C. (2024). Superhero pretense, superhero identification, and risk-taking in preschool-aged children. J. Pediatr. Psychol. 49, 234–243. 10.1093/jpepsy/jsad045 37478354

[B40] Muller J. N. Moroco A. Loloi J. Portolese A. Wakefield B. H. King T. S.. (2020). Violence depicted in superhero-based films stratified by protagonist/antagonist and gender. Cureus 12:e6980. 10.7759/cureus.6843 32181080 PMC7053689

[B41] O'Connel L. (2019). Top-ranked children's Halloween costumes in the United States in 2019. Statista. Availabe online at: https://www.statista.com/statistics/922593/most-popular-halloween-costumes-for-children-us/ (accessed August 5, 2024).

[B42] Ostrov J. M. Kamper K. E. Hart E. J. Godleski S. A. Blakely-McClure S. J. (2014). A gender-balanced approach to the study of peer victimization and aggression subtypes in early childhood. Dev. Psychopathol. 26, 575–587. 10.1017/S0954579414000248 25047285

[B43] Padilla-Walker L. M. Carlo G. (Eds.) (2014). Prosocial Development: A Multidimensional Approach. Oxford: Oxford University Press.

[B44] Ramdaeni S. Adriany V. Yulindrasari H. (2020). “Gender and toys in early childhood education,” in International Conference on Early Childhood Education and Parenting 2019 (ECEP 2019) (Dordrecht: Atlantis Press), 250–254. 10.2991/assehr.k.200808.049

[B45] Reschke P. J. Fraser A. M. Pickett J. Workman K. Lehnardt H. Stockdale L. A.. (2023). Variability in infant helping and sharing behaviors across the second and third years of life: differential roles of target and socialization. Dev. Psychol. 59, 524–538. 10.1037/dev0001441 36074587

[B46] Rideout V. Robb M. B. (2020). The Common Sense Census: Media Use by Kids Age Zero to Eight, 2020. San Francisco, CA: Common Sense Media.

[B47] Rubin L. Livesay H. (2006). Look, up in the sky! Using superheroes in play therapy. Int. J. Play Ther. 15, 117–133. 10.1037/h0088911

[B48] Salmivalli C. Lagerspetz K. Björkqvist K. Österman K. Kaukiainen A. (1996). Bullying as a group process: participant roles and their relations to social status within the group. Aggress. Behav. 22, 1–15. 10.1002/(SICI)1098-2337(1996)22:1<1::AID-AB1>3.0.CO;2-T

[B49] Salmivalli C. Voeten M. Poskiparta E. (2011). Bystanders matter: associations between reinforcing, defending, and the frequency of bullying behavior in classrooms. J. Clin. Child Adolesc. Psychol. 40, 668–676. 10.1080/15374416.2011.597090 21916686

[B50] Sandberg D. E. Meyer-Bahlburg H. F. (1994). Variability in middle childhood play behavior: effects of gender, age, and family background. Arch. Sex. Behav. 23, 645–663. 7872860 10.1007/BF01541817

[B51] Shawcroft J. Coyne S. M. (2022). Does Thor ask iron man for help? Examining help-seeking behaviors in Marvel superheroes. Sex Roles 87, 223–236. 10.1007/s11199-022-01301-5

[B52] Shawcroft J. Gale M. Coyne S. M. Rogers A. A. Austin S. Holmgren H. G.. (2024). Ariel, Aurora, or Anna? Disney princess body size as a predictor of body esteem and gendered play in early childhood. Psychol. Pop. Media 13, 591–602. 10.1037/ppm0000494

[B53] Thornberg R. Jungert T. (2013). Bystander behavior in bullying situations: basic moral sensitivity, moral disengagement, and defender self-efficacy. J. Adolesc. 36, 475–483. 10.1016/j.adolescence.2013.02.003 23522703

[B54] Tremblay R. E. (2000). The development of aggressive behaviour during childhood: what have we learned in the past century? Int. J. Behav. Dev. 24, 129–141. 10.1080/016502500383232

[B55] van Noorden T. H. J. Haselager G. J. T. Cillessen A. H. N. Bukowski W. M. (2014). Empathy and involvement in bullying in children and adolescents: a systematic review. J. Youth Adolesc. 44, 637–657. 10.1007/s10964-014-0135-6 24894581

[B56] Vandewater E. A. Lee S. J. (2009). Measuring children's media use in the digital age: issues and challenges. Am. Behav. Sci. 52, 1152–1176. 10.1177/0002764209331539 19763246 PMC2745155

[B57] Vygotsky L. S. (1978). Mind in Society: The Development of Higher Psychological Processes. London: Harvard University Press.

[B58] Wang Y. Vickery N. Tarlinton D. Ploderer B. Knight L. Blackler A.. (2022). “Exploring the affordances of digital toys for young children's active play,” in Proceedings of the 34th Australian Conference on Human-Computer Interaction (Canberra: ACM), 187–197.

